# ‘Not at target’: prevalence and consequences of inadequate disease control in systemic lupus erythematosus—a multinational observational cohort study

**DOI:** 10.1186/s13075-022-02756-3

**Published:** 2022-03-14

**Authors:** Rangi Kandane-Rathnayake, Worawit Louthrenoo, Alberta Hoi, Shue-Fen Luo, Yeong-Jian J. Wu, Yi-Hsing Chen, Jiacai Cho, Aisha Lateef, Laniyati Hamijoyo, Sandra V. Navarra, Leonid Zamora, Sargunan Sockalingam, Yuan An, Zhanguo Li, Yasuhiro Katsumata, Masayoshi Harigai, Yanjie Hao, Zhuoli Zhang, Jun Kikuchi, Tsutomu Takeuchi, B. M. D. B. Basnayake, Madelynn Chan, Kristine Pek Ling Ng, Nicola Tugnet, Sunil Kumar, Shereen Oon, Fiona Goldblatt, Sean O’Neill, Kathryn A. Gibson, Naoaki Ohkubo, Yoshiya Tanaka, Sang-Cheol Bae, Chak Sing Lau, Mandana Nikpour, Vera Golder, Eric F. Morand, For the Asia-Pacific Lupus Collaboration

**Affiliations:** 1 Monash Medical Centre, School of Clinical Sciences, Monash University, 246 Clayton Road, Clayton, VIC 3168 Australia; 2 Chiang Mai University, Chiang Mai, Thailand; 3 Chang Gung Memorial Hospital, Taoyuan County and Keelung, Taiwan; 4 Taichung Veterans General Hospital, Taichung, Taiwan; 5 National University Hospital, Singapore, Singapore; 6 University of Padjadjaran, Bandung, Indonesia; 7 University of Santo Tomas Hospital, Manila, Philippines; 8 University of Malaya, Kuala Lumpur, Malaysia; 9 People’s Hospital Peking University Health Sciences Centre, Beijing, China; 10 Tokyo Women’s Medical University, Tokyo, Japan; 11 Peking University First Hospital, Beijing, China; 12 Keio University, Tokyo, Japan; 13 Teaching Hospital, Kandy, Sri Lanka; 14 Tan Tock Seng Hospital, Singapore, Singapore; 15 Waitemata District Health Board, Auckland, New Zealand; 16 Auckland District Health Board, Auckland, New Zealand; 17 Middlemore Hospital, Auckland, New Zealand; 18 The University of Melbourne at St Vincent’s Hospital, Fitzroy, Victoria Australia; 19 Royal Adelaide Hospital and Flinders Medical Centre, Adelaide, Australia; 20 University of New South Wales and Ingham Institute of Applied Medical Research, Liverpool, Australia; 21 Eli Lilly Pty Ltd. Australia, Liverpool Hospital, Sydney, Australia; 22 University of Occupational and Environmental Health, Kitakyushu, Japan; 23 Hanyang University Hospital for Rheumatic Diseases, Seoul, South Korea; 24 University of Hong Kong, Pok Fu Lam, Hong Kong

**Keywords:** Systemic lupus erythematosus, Disease activity, Outcomes, Quality of life, Unmet need

## Abstract

**Background:**

The unmet need in systemic lupus erythematosus (SLE) with the current standard of care is widely recognised, but few studies have quantified this. The recent definition of treat-to-target endpoints and other thresholds of uncontrolled disease activity provide an opportunity to formally define unmet need in SLE. In this study, we enumerated the prevalence of these states and examined their association with adverse outcomes.

**Methods:**

Data were collected prospectively in a 13-country longitudinal SLE cohort between 2013 and 2019. Unmet need was defined as never attaining lupus low disease activity state (LLDAS), a time-adjusted mean SLEDAI-2K (AMS) > 4, or ever experiencing high disease activity status (HDAS; SLEDAI-2K ≥10). Health-related quality of life (HRQoL) was assessed using SF36 (v2) and damage accrual using the SLICC-ACR SLE Damage Index (SDI).

**Results:**

A total of 3384 SLE patients were followed over 30,313 visits (median [IQR] follow-up 2.4 [0.4, 4.3] years). Eight hundred thirteen patients (24%) never achieved LLDAS. Median AMS was 3.0 [1.4, 4.9]; 34% of patients had AMS > 4. Twenty-five per cent of patients had episodes of HDAS. Each of LLDAS-never, AMS>4, and HDAS-ever was strongly associated with damage accrual, higher glucocorticoid use, and worse HRQoL. Mortality was significantly increased in LLDAS-never (adjusted HR [95% *CI*] = 4.98 [2.07, 12.0], *p*<0.001) and HDAS-ever (adjusted hazard ratio (HR) [95% *CI*] = 5.45 [2.75, 10.8], *p*<0.001) patients.

**Conclusion:**

Failure to achieve LLDAS, high average disease activity, and episodes of HDAS were prevalent in SLE and were significantly associated with poor outcomes including organ damage, glucocorticoid exposure, poor quality of life, and mortality.

**Supplementary Information:**

The online version contains supplementary material available at 10.1186/s13075-022-02756-3.

## Background

Systemic lupus erythematosus (SLE, or lupus) is characterised by recurrent immune-mediated inflammatory damage in multiple organ systems [1], resulting in a marked loss of life expectancy [2] and among the top 10 causes of death in young women in the USA [3]. SLE treatment has changed little in the past 50 years due to the paucity of approved or reimbursed novel therapies [4]. As a result, the majority of patients are still treated with non-specific agents including glucocorticoids, which can contribute to harmful long-term outcomes that include irreversible organ damage [5]. The failure to improve SLE outcomes is in stark contrast to paradigm changes in outcomes in other autoimmune diseases such as rheumatoid arthritis (RA). Transformation of patient outcomes in RA began with the recognition that historical standards of care were associated with poor outcomes, followed by treat-to-target strategies driven by validated thresholds of inadequate response. While patient outcomes in RA, including mortality, have been transformed in the last 20 years in response to these approaches [6], there has been no such improvement in SLE in the same period [7].

To date, only two targeted therapy has received regulatory approval for SLE treatment. Belimumab, a monoclonal antibody (mAb) targeted against the B cell activating factor (BAFF) [8], was approved in the USA in 2011 for active SLE [9] and lupus nephritis [10], but uptake has been low in many care settings including in the USA [11]. Very recently, anifrolumab, a type 1 interferon receptor antagonist, received FDA approval for the treatment of SLE [12, 13]. As more advanced therapies for SLE emerge, the proportion of SLE patients whose disease characteristics identify them as having unmet need that might justify such therapies is unknown. Identifying the proportion of SLE patients who could benefit from advanced therapies could assist physicians, hospitals, and regulators to plan for their use, help patient groups lobby for reimbursement of SLE drugs, and plug gaps in understanding in the wider medical community about the needed changes in SLE treatment.

A limiting factor on quantifying unmet need in SLE has been a lack of formal definitions. The recent prospective validation of the Lupus Low Disease Activity State (LLDAS) [14] as affording time-dependent protection from adverse outcomes, findings confirmed for both damage accrual and mortality in independent studies [15, 16], indicates that failure to attain LLDAS is an undesirable disease state. Other measures of inadequate disease control have also recently emerged, which allow the enumeration of proportions of patients with indicators consistent with inadequate disease control, and who therefore have the potential to benefit from treatment advances.

## Methods

### Aim

In this study, we aimed to evaluate the prevalence, and consequences, of potential unmet need in SLE, using recently defined descriptors of inadequate disease control.

### Study population

Data from the Asia Pacific Lupus Collaboration (APLC) patient cohort, collected prospectively between 2013 and 2019, were used to conduct this study. Patients were recruited from 23 sites across 13 countries. All patients met either the 1997 American College of Rheumatology (ACR) Modified Classification Criteria for SLE [17] or the Systemic Lupus International Collaborating Clinics (SLICC) 2012 Classification Criteria [18] and provided written informed consent [19].

### Data collection

Data were collected during routine patient follow-up visits using standardised electronic or paper data-collection forms. The minimum prescribed visit frequency was 6 months, with the majority of patients having more frequent visits based on clinical need.

Baseline demographic data were collected at enrolment and included age, gender, self-reported ethnicity, date of onset of SLE, date of SLE diagnosis, smoking status, highest education level, and family history. At each visit, SLE Disease Activity Index (SLEDAI)-2K [20], SELENA-SLEDAI flare index [21], and physician global assessment (PGA 0–3) [22]), and data on all medications and doses, were collected. Organ damage was measured at baseline and annually using the SLICC-ACR Damage Index (SDI) [23]; a change of one unit in SDI has been demonstrated to be clinically significant [24] and was chosen to define damage accrual. Health-related quality of life (HRQoL) was captured using the Short Form 36 (v2) (SF36) and expressed as mental and physical component summary (MCS and PCS, respectively) scores, as described [25].

### Definitions of unmet need

LLDAS was defined on a per-visit basis according to the recently validated definition of Golder et al. [14]. Briefly, this includes the requirement for all of SLEDAI-2K ≤ 4, excluding major organ activity, absence of new SLEDAI-2K activity compared to the preceding visit, PGA ≤ 1 (0–3), and daily prednisolone dose ≤ 7.5 mg/day; anti-malarials and immunosuppressant use are permitted. A time-adjusted mean SLEDAI-2K (AMS) was calculated as a measure of disease activity over time [26], and time-adjusted mean PGA and prednisolone dose similarly calculated. High disease activity status (HDAS; SLEDAI-2K ≥10) was documented as described [27, 28]. Inadequate disease control was classified as follows: patients never achieving LLDAS during the period of observation (LLDAS-never); persistent active disease defined as AMS > 4, meaning that on average the SLEDAI-2K was always above 4 throughout the period of observation; or exhibiting HDAS at any time (HDAS-ever) [27, 28].

### Statistical analysis

Statistical analyses were performed using Stata V. 15.1 (StataCorp, College Station, TX, USA). Continuous variables were described as median and interquartile range (IQR) due to the skewed nature of the data and compared using Wilcoxon rank-sum tests. Categorical variables were described as frequency (%) and compared using *χ*^2^ tests. In all analyses, a *p*-value ≤0.05 was considered statistically significant.

Survival (time-to-event) analyses, i.e. Cox regression models, were used to examine the associations of unmet need definitions with damage accrual and mortality. When performing survival analyses with damage accrual, we incorporated Prentice, Williams and Peterson modelling with gap time (PWP-GT) to set up the data to allow multiple ‘failures’ per patient as some patients accrued damage more than once during the study observation period. In addition, clustering was specified in these Cox regression models to account for intragroup correlation. The results from the time-dependent Cox regression analyses are presented as hazard ratios with corresponding 95% confidence intervals (CI).

Furthermore, generalised estimating equations (GEE) methods were used to examine the associations of unmet need definitions with prednisolone dose and HRQoL assessed using SF36. These outcomes were captured as continuous variables; therefore, GEE models were specified for Gaussian distribution, identity link, and exchangeable correlation matrix. Regression coefficients estimated from GEE analyses represented the change in estimated population averages, i.e. mean changes, which we reported with corresponding 95% CI.

## Results

We studied 3384 SLE patients who were followed over median [IQR] 2.4 [0.4, 4.3] years, comprising 30,313 visits. The median [IQR] age at enrolment was 39 [30, 50], 3109 (92%) of patients were female, and the majority were of Asian ethnicity (Table 1).

**Table 1:** Patient characteristics

	Summary statistics
	***n*** = 3384
**Demographics**	
Age at enrolment (years), *median [IQR] (range)*	39 [30, 50] (17,87)
Age at diagnosis (years), *median [IQR] (range)*	29 [21, 39] (1, 84)
Disease duration at enrolment (years), *median [IQR] (range)*	8 [3, 15] (0, 51)
Study duration (years), *median [IQR] (range)*	2.4 [0.4, 4.3] (0, 7)
Total visits, *median [IQR] (range)*	7 [2, 12] (1, 81)
Female, *n (%)*	3109 (92)
Family history of SLE, *n (%)*	249 (7.9)
Asian ethnicity, *n (%)*	2868 (87.4)
Current smoker at enrolment, *n (%)*	162 (5.1)
Tertiary education, *n (%)*	1482 (49.1)
Serological profile at enrolment, *n (%)*	
ANA positivity	2939 (87.5)
Anti-dsDNA positivity	2430 (72.4)
Low complement	2116 (63.0)
**Medication use-ever (at least once)**, *n (%)*	
Prednisolone (PNL) use	2765 (81.7)
TAM-prednisolone (mg/day), *median [IQR] (range)*	5.0 [2.2, 9.1] (0, 50)
Anti-malarial (AM) use	2577 (76.2)
Immunosuppressant (IS) use	1751 (51.7)
Biological use	35 (1.03)
**Clinical profile across the follow-up period**	
LLDAS ever, *n (%)*	2529 (75.7)
LLDAS never, *n (%)*	813 (24.3)
Percent time spent in LLDAS, *median [IQR] (range)*	45.8 [8.4, 73.8] (0, 100)
TAM-PGA, *median [IQR] (range)*	0.43 [0.22, 0.75] (0, 3)
AMS, *median [IQR] (range)*	3.0 [1.4, 4.8] (0, 22)
AMS > 4, *n (%)*	932 (33.8)
High Disease Activity State (HDAS; SLEDAI ≥ 10) ever, *n (%)*	851 (25.1)
Any flare (mild/moderate/severe) ever, *n (%)*	1762 (52.1)
Organ damage present at recruitment, *n (%)*	1165 (37.9)
Damage accrual during the study period, *n (%)*	561 (18.3)
Deaths, *n (%)*	58 (1.7)
No. of patients with at least one SF36 survey, *n (%)*	2,583 (76.3)
TAM-SF36 (PCS), *median [IQR] (range)*	49.2 [43.7, 53.5] (19.7, 62.3)
TAM-SF36 (MCS), *median [IQR] (range)*	49.2 [42.6, 53.7] (18.8, 63.7)

*IQR* interquartile range, *TAM* time-adjusted mean, *AMS* TAM-SLEDAI-2K, *PGA* physician global assessment of disease activity (0–3), *LLDAS* lupus low disease activity state, *HDAS* high disease activity status (SLEDAI-2K ≥ 10), *SA* serological activity, *GC* glucocorticoid use, *AM* hydroxychloroquine/chloroquine, *IS* mycophenolate/mycophenolic acid/azathioprine/cyclosporine/methotrexate/tacrolimus/leflunomide/cyclophoamide, *biologics* rituximab/belimumab, *SF36* Short Form 36 (v2), *PCS* physical component summary, *MCS* mental component summary

**Fig. 1:**
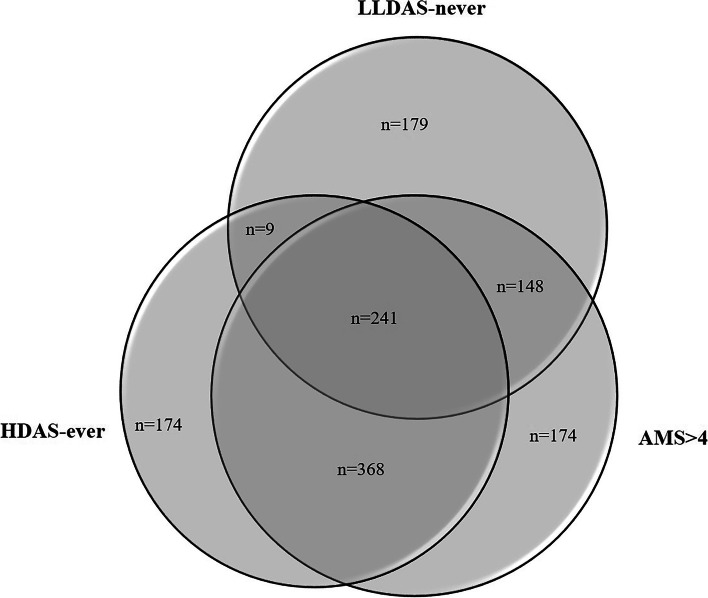
A total of 3384 SLE patients were followed over median 2.4 years, comprising 30,313 visits. Venn diagram depicting numbers of patients who, during this observation period, did not attain lupus low disease activity state (LLDAS-never), had persistently active disease (AMS ≥ 4), or had at least one episode of high disease activity state (HDAS-ever), or combinations of these states

We first assessed unmet need defined by non-attainment of LLDAS. LLDAS status was not determined for 2.6% of visits, due predominantly to missing PGA or incomplete SLEDAI-2K. Overall, patients were in LLDAS in 13,447/28,760 visits (47%), and consequently, not in LLDAS in 15,223 visits (53%), and the median percentage of observed time each patient spent in LLDAS was 45.8% [IQR 8.5, 73.8] (range: 0, 100) (Table 1). Eight hundred thirteen patients (24.3%) never achieved LLDAS during the observation period (LLDAS-never). Two hundred forty-one patients (7%) met all three definitions (Fig. 1).

Characteristics of patients stratified by LLDAS attainment are shown in Table 2. Compared to patients who ever attained LLDAS, patients who never achieved LLDAS were younger, more likely to be of Asian ancestry, and had shorter median follow-up (Table 2). Compared to patients who ever achieved LLDAS, LLDAS-never was associated with significantly higher disease activity over time measured by SLEDAI-2K or PGA, higher glucocorticoid dosing, and increased mortality, as well as significantly lower HRQoL measured by both MCS and PCS (Table 2).

**Table 2:** Patient characteristics by definitions of disease state

	LLDAS			AMS			HDAS		
	Ever	Never	***p***-value	≤4	>4	***p***-value	Never	Ever	***p***-value
	***n***=2529	*n*=813		***n***=1829	***n***=932		***n***=2533	***n***=851	
**Demographics**									
Age at enrolment (years), *median [IQR]*	40 [31, 51]	37 [29, 48]	<0.001	41 [31, 52]	35 [27, 45]	<0.001	42 [32, 52]	34 [26, 44]	<0.001
Age at diagnosis (years), *median [IQR]*	29 [22, 40]	27 [21, 36]	<0.001	30.5 [23, 41]	26 [20, 34]	<0.001	30 [22, 40]	26 [20, 34]	<0.001
Disease duration (years), *median [IQR]*	8 [3, 15]	8 [3, 14]	0.38	7 [3, 14]	7 [3, 13]	0.5	9 [3, 16]	6 [2, 12]	<0.001
Study duration (years), *median [IQR]*	2.9 [1, 4.8]	1 [0, 2.9]	<0.001	3.0 [1.6, 4.7]	3.1 [1.7, 4.9]	0.09	2.0 [0.2, 4]	3.3 [1.7, 5.1]	<0.001
Total visits, *median [IQR]*	9 [4, 15]	4 [1, 9]	<0.001	9 [5, 14]	9 [5, 15]	0.02	6 [2, 10]	10 [6, 19]	<0.001
Females, *n (%)*	2324 (91.9)	747 (92.3)	0.7	1698 (92.8)	865 (92.8)	0.9	2314 (91.5)	796 (93.5)	0.059
Asian ethnicity, *n (%)*	2129 (86.1)	719 (91.6)	<0.001	1541 (85.7)	822 (89.1)	0.013	2121 (87.1)	747 (88.3)	0.4
**Serological profile at enrolment,** ***n (%)***									
ANA positivity	2235 (89.0)	673 (83.1)	<0.001	1699 (93.3)	847 (91.1)	0.028	2155 (86.0)	784 (92.1)	<0.001
Anti-dsDNA positivity	1807 (71.9)	599 (74.0)	0.3	1314 (72.4)	798 (85.7)	<0.001	1734 (69.2)	696 (81.8)	<0.001
Low complement	1567 (62.4)	527 (65.1)	0.17	1159 (63.9)	680 (73.0)	<0.001	1527 (60.9)	589 (69.1)	<0.001
**Clinical profile,** ***n (%)***									
AMS, *median [IQR]*	2.5 [1.1, 4.0]	5.5 [3.7, 8.0]	<0.001	2.0 [0.7, 3.0]	5.9 [4.8, 7.7]	<0.001	2.1 [0.9, 3.7]	5.8 [4.2, 7.9]	<0.001
TAM PGA, *median [IQR]*	0.4 [0.2, 0.6]	0.9 [0.5, 1.2]	<0.001	0.3 [0.1, 0.5]	0.8 [0.5, 1.1]	<0.001	0.3 [0.2, 0.6]	0.7 [0.4, 1.0]	<0.001
TAM PNL, *median [IQR]*	4.6 [1.5, 7.3]	10.1 [8, 15]	<0.001	4.3 [0.9, 7.5]	8.3 [5, 12.2]	<0.001	4.6 [1.0, 7.5]	8.3 [5, 12.3]	<0.001
Percent time in LLDAS, *median [IQR]*	55.6 [33.3, 81]	0	<0.001	60.4 [34.1, 85]	11.0 [0, 34]	<0.001	55.5 [20.5, 83]	18.8 [0, 44]	<0.001
Flare (mild/moderate/severe) ever	1352 (53.4)	410 (50.4)	0.14	948 (51.8)	784 (84.1)	<0.001	1022 (40.3)	740 (87.0)	<0.001
Organ damage present at recruitment (SDI > 0)	910 (39.3)	318 (44.2)	0.018	622 (34.6)	348 (37.9)	0.084	857 (38.1)	308 (37.5)	0.8
Damage accrual during the study period (change in SDI > 0)	380 (16.4)	104 (14.5)	0.2	326 (18.1)	235 (25.6)	<0.001	345 (15.3)	216 (26.3)	<0.001
Deaths	27 (1.1)	31 (3.8)	<0.001	19 (1.0)	31 (3.3)	<0.001	23 (0.9)	35 (4.1)	<0.001
**SF36,** ***median [IQR]***									
TAM PCS	49.5 [44, 56]	48.4 [42, 52]	0.009	49.7 [44, 54]	48.4 [43, 53]	0.003	49.8 [44, 54]	48.1 [43, 52]	<0.001
TAM MCS	49.7 [44, 54]	46.1 [39, 52]	<0.001	49.7 [43, 54]	48.1 [42, 53]	<0.001	49.7 [43, 54]	48.1 [42, 53]	<0.001

*p*-values were calculated using Wilcoxon rank-sum tests for median comparisons and chi-squared tests for proportion comparison. *IQR* interquartile range, *AMS* time-adjusted mean SLEDAI-2K, *PGA* physician global assessment of disease activity (0–3), *PNL* prednisolone, *TAM* time-adjusted mean, *LLDAS* lupus low disease activity state, *SDI* SLICC-ACR Damage Index, *HDAS* high disease activity status, *SA* serological activity, *GC* glucocorticoid use, *SF36* Short Form 36 (v2)

We next assessed a definition of persistent active disease over time, defined as AMS > 4, meaning on average a patient’s SLEDAAI-2K was above 4 throughout the period of observation. The median AMS in the cohort was 3.0 [IQR 1.4, 4.9] (range: 0, 22) and 932 patients (34%) had AMS > 4 (Table 1). Compared to patients with AMS ≤4, patients with AMS > 4 were younger and more likely to be Asian and were more likely to have serological activity (Table 2). In terms of outcomes, AMS > 4 was associated with significantly higher glucocorticoid dosing, more flares, more damage accrual, and higher mortality, as well as significantly less time in LLDAS and lower HRQoL measured by both MCS and PCS (Table 2).

HDAS (SLEDAI-2K ≥ 10) has recently been defined as a state associated with worse outcomes in SLE even if only experienced once [27, 28]. Eight hundred fifty-one patients (25%) had HDAS at least once during the study period, accounting for 8% (*n*=2418) of all visits. Compared to patients who never experienced HDAS during the period of observation, HDAS-ever patients were younger and had a more recent onset of disease and increased serological activity, but HDAS was not associated with ethnicity (Table 2). In terms of disease outcomes, HDAS-ever was associated with higher disease activity across the observation period measured by SLEDAI-2K or PGA, higher glucocorticoid doses, more flares, less time in LLDAS, and higher rates of both damage accrual and death (Table 2). In addition, HRQoL (MCS and PCS) was lower in HDAS-ever patients (Table 2).

Each of LLDAS-never, AMS>4, and HDAS-ever was strongly associated with poor outcomes over time, including more damage accrual, higher risk of mortality, high glucocorticoid use, and worse SF36 PCS and MCS scores reflecting poor HRQoL (Table 3 and Supplementary tables S1, S2, S3, S4, S5). For example, never attaining LLDAS was associated with a near-5-fold risk of death after adjustment for confounders (Table 3 and Supplementary Table S1). In the HDAS group, the instantaneous risk (hazard) of mortality was more than five times that of patients without HDAS after adjustment for confounders (adjusted *HR* 5.45 (2.75,10.80), *p*<0.001) (Table 3 and Supplementary Table S2).

**Table 3:** Longitudinal associations of outcomes with different SLE unmet need definitions

Outcomes	LLDAS-never	AMS > 4	HDAS-ever
	**HR**^**1**^ **(95%** ***CI*),** ***p*-value**	**HR**^**1**^ **(95%** ***CI*),** ***p*-value**	**HR**^**1**^ **(95%** ***CI*),** ***p*-value**
**Damage accrual**			
Unadjusted	1.52 (1.31, 1.76), *p* < 0.001	1.38 (1.18, 1.61), *p* < 0.001	1.85 (1.47, 2.31), *p* < 0.001
Adjusted^a^	1.46 (1.26, 1.69), *p* < 0.001	1.36 (1.16, 1.59), *p* < 0.001	1.81 (1.43, 2.30), *p* < 0.001
**Mortality**			
Unadjusted	6.64 (2.83, 15.6), *p* < 0.001	2.99 (1.68, 5.3), *p* < 0.001	6.97 (3.82, 12.7), *p* < 0.001
Adjusted^b^	4.98 (2.07, 12.0), *p* < 0.001	2.36 (1.29, 4.33), *p* = 0.006	5.45 (2.75, 10.80), *p* < 0.001
	**RC**^**2**^ **(95%** ***CI*),** ***p*-value**	**RC**^**2**^ **(95%** ***CI*),** ***p*-value**	**RC**^**2**^ **(95%** ***CI*),** ***p*-value**
**Cumulative prednisolone (PNL)**			
Unadjusted	5.61 (5.34, 5.88), *p* < 0.001	4.08 (3.66, 4.51), *p* < 0.001	8.96 (8.02, 9.91), *p* < 0.001
Adjusted^c^	5.71 (5.38, 6.03), *p* < 0.001	3.39 (2.95, 3.83), *p* < 0.001	9.04 (7.80, 10.3), *p* < 0.001
**TAM-PNL at visit**			
Unadjusted	1.25 (1.08, 1.41), *p* < 0.001	2.33 (1.90, 2.76), *p* < 0.001	1.18 (0.88, 1.47), *p* < 0.001
Adjusted^c^	1.35 (1.17, 1.52), *p* < 0.001	2.52 (2.20, 2.85), *p* < 0.001	1.41 (0.88, 1.94), *p* < 0.001
**PCS**			
Unadjusted	−1.59 (−1.90, −1.28), *p* < 0.001	−1.04 (−1.58,−0.50), *p* < 0.001	−2.49 (−3.07, −1.90), *p* < 0.001
Adjusted^d^	−1.40 (−1.71, −1.09), *p* < 0.001	−0.96 (−1.50,−0.43), *p* < 0.001	−2.17 (−2.78, −1.57), *p* < 0.001
**MCS**			
Unadjusted	−1.22 (−1.58, −0.85), *p* < 0.001	−0.84 (−1.42, −0.27), *p* < 0.001	−1.37 (−2.03, −0.70), *p* < 0.001
Adjusted^e^	−1.20 (−1.57, −0.84), *p* < 0.001	−0.97 (−1.56, −0.38), *p* = 0.001	−1.29 (−1.96, −0.63), *p* < 0.001

^1^Hazard ratios (HR) derived using Cox regression analyses. ^2^Regression coefficients (RCs) derived using generalised estimating equations (GEE). RC indicates the mean difference between the unmet need definition and the corresponding comparator (e.g. not in LLDAS vs. in LLDAS). TAM-PNL at visit = time-adjusted mean prednisolone since the baseline visit to each routine visit

^a^HRs adjusted for age, disease duration, Asian ethnicity, tertiary education, and cumulative PNL. Full multivariable models are presented in Supplementary table S1

^b^HRs adjusted for cumulative PNL and ACR/SLICC SDI score. Full multivariable models are presented in Supplementary table S2

^c^RCs adjusted for age, disease duration, Asian ethnicity, presence of flare, and ACR/SLIC SDI score. Full multivariable models for prednisolone are presented in Supplementary table S3

^d^RCs adjusted for age, disease duration, Asian ethnicity, tertiary education, cumulative PNL, presence of flare, and organ damage. Full multivariable models are presented in Supplementary table S4

^e^RCs adjusted for Asian ethnicity, tertiary education, and cumulative PNL. Full multivariable models are presented in Supplementary table S5

The use of standard of care medications in the unmet need patient population was also evaluated. As SLE treatments change over time in individual patients, these data were analysed on a per-visit basis. Unmet need status did not appear to be due to under-treatment. For example, over 70% of LLDAS-never patients were receiving combination therapy with at least two of anti-malarials, glucocorticoids, and immunosuppressants, and fewer than 10% were on no treatment or anti-malarial monotherapy (Table 4). Similar profiles of medication use were observed for the other definitions (Table 4).

**Table 4:** Medication use, stratified by patient visits meeting unmet need definitions

Medications (therapy)	All visits Total = 30,313 ***n*** (%)	LLDAS-never Total = 15,223 ***n*** (%)	AMS > 4 Total = 7925 ***n*** (%)	HDAS-ever Total = 2418 ***n*** (%)
No therapy	1553 (5.12)	383 (2.52)	189 (2.38)	39 (1.61)
Monotherapy	9167 (30.2)	4015 (26.4)	2014 (25.4)	515 (21.3)
*PNL alone*	*5222 (17.2)*	*2816 (18.5)*	*1391 (17.6)*	*412 (17.0)*
*AM alone*	*3567 (11.8)*	*1034 (6.79)*	*543 (6.85)*	*81 (3.35)*
*IS alone*	*378 (1.25)*	*165 (1.08)*	*80 (1.01)*	*22 (0.91)*
Dual therapy	12,935 (42.7)	6957 (45.7)	3604 (45.5)	1237 (51.2)
*PNL+AM*	*7902 (26.1)*	*4270 (28.1)*	*2281 (28.8)*	*841 (34.8)*
*PNL+IS*	*3610 (11.9)*	*2224 (14.6)*	*1079 (13.6)*	*369 (15.3)*
*AM+IS*	*1423 (4.7)*	*463 (3.04)*	*244 (3.08)*	*27 (1.12)*
Triple therapy (PNL+AM+IS)	6658 (22)	3868 (25.4)	2118 (26.7)	627 (25.9)
**Combination therapy (dual/triple)**	**19,593 (64.6)**	**10,825 (71.1)**	**5722 (72.2)**	**1864 (77.1)**

## Discussion

The standard of care for SLE has changed little in recent decades, with only two new therapy approved for active SLE in the last 50 years. Accompanying this paucity of treatment innovation, improvements in SLE mortality observed in the late twentieth century have plateaued in the first decades of the twenty-first century [7]. This is in contrast to outcomes in RA, which have dramatically improved in the same period [6]. The ability to harness new therapies in treat-to-target approaches to SLE management, as recently advocated [4, 29, 30], requires additions to the current knowledge base. Ultimately, formal treat-to-target studies should be performed, using failure to attain well-validated endpoints to trigger treatment escalation, and using long-term harm as the outcome measure. Before this, however, access to such treatments is required, and understanding the extent of unmet need is needed in order to inform physicians and regulators. Here, we have used recently defined and/or validated indices of inadequate disease control, and a large multinational longitudinal SLE cohort, to evaluate the extent and consequences of unmet need in SLE patients receiving standard of care.

Our findings demonstrate that unmet need in SLE is prevalent and is associated with poor outcomes. In this large dataset, half of patients’ observed time overall was spent not in LLDAS, and a quarter of patients did not attain LLDAS at any time across the period of observation. In turn, non-attainment of LLDAS was associated with higher overall disease activity and glucocorticoid exposure, as well as more damage accrual and lower HRQoL. Strikingly, never attaining LLDAS was associated with a near fivefold increase in mortality. LLDAS attainment has been shown in multiple retrospective cohort studies, and recently in a prospective multicentre study, to be associated with protection from SLE flare, damage accrual, and death, and to be associated with improved HRQoL [14–16, 25], and where studied these associations are dose-dependent, i.e. less time in LLDAS is associated with poorer outcomes [14, 31]. While the associations of never attaining LLDAS with worse outcomes were therefore expected, the observation that a quarter of patients did not attain LLDAS on even a single occasion signifies that this treat to target goal is insufficiently frequently met with the current standard of care.

Other definitions of potential unmet need showed similar findings. A SLEDAI-2K of greater than four is regarded as indicating active disease. We used AMS > 4 as a measure of poor control of disease activity; patients with AMS > 4 had an average SLEDAI-2K > 4 over the entire period of observation. One-third of patients had AMS > 4, consistent with enduringly poorly controlled disease activity across the period of observation, and this state was associated with increased flares, glucocorticoid use, mortality, and damage accrual, as well as lower HRQoL. Another simple index of poor disease control, HDAS, recently was demonstrated to be associated with poor outcomes in SLE observational cohorts even if only exhibited once during a period of observation [27, 28]. HDAS was observed at least once in a quarter of all patients, and the association of this state on even a single occasion spanned the same adverse outcomes as AMS>4, including a strong association with mortality. This suggests that even single episodes of HDAS are associated with an adverse impact on patient outcomes, a finding similar to that recently reported in a single-centre cohort study [28], and that these single episodes are comparable in outcome to persistently active disease at a lower threshold. Interestingly, all the definitions of unmet need tested here were also associated with younger age of disease onset; this may have parallels in the observation that earlier disease onset in SLE is linked with higher genetic risk scores and worse outcomes [32].

These data using empirical cut-offs demonstrate formally that many SLE patients remain inadequately controlled in the setting of current standards of care. This raises the question of whether treatments could improve outcomes for patients so identified. Of note, we have recently described an algorithm for identifying HDAS episodes from SLE patient medical records without the need for SLEDAI-2K scores [27]. Such patients may be more likely to respond to targeted interventions. Increased responses to therapy with atacicept were seen in SLE patients with HDAS at baseline [33], and the same SLEDAI cut-off (≥10) which defines HDAS was associated with increased likelihood of response to treatment with belimumab in post hoc analysis [34]. This same analysis by Van Vollenhoven et al. also showed associations of response to belimumab with serological activity, i.e. low complement and/or anti-dsDNA, and glucocorticoid treatment [34]. We analysed the combination of these factors, i.e. HDAS, serological activity, and glucocorticoid treatment, and found that 23.1% of patients met these criteria at least once, and doing so was associated with similar if not greater associations with poor outcomes such as damage accrual, low quality of life, and mortality (data not shown). Although it is possible that these findings relate less to responsiveness per se than to the ability to measure a response using existing trial endpoints [35], identifying patients who exhibit metrics consistent with unmet need may enable targeting for treatment escalation or enrolment in clinical trials.

There are several limitations to the interpretation of this study. First, the research question was designed and data analysed retrospectively, although data were collected prospectively using standardised data collection forms. Secondly, no inference about the potential response to an intervention in patients who meet these unmet need criteria can be drawn in the absence of a study of such an intervention. Thirdly, this multicentre study was performed in the Asia-Pacific region in a cohort of majority with Asian ancestry and variations in health systems including access to biologicals that may impact our results, although the patterns of medication use were broadly similar to those reported in other cohorts.

## Conclusion

In conclusion, this study of a large multicentre cohort indicates a high prevalence of unmet need in SLE, defined using empirical thresholds, and that the consequences of these states of unmet need include increased damage, increased mortality, and reduced quality of life. Unmet need in SLE is a prevalent and serious issue, and improved therapeutic strategies are urgently needed.

## Supplementary Information

**Additional file 1: Supplementary Table S1.** Associations of SLE unmet need definitions with organ damage accrual, adjusted for other potential confounding factors.

**Additional file 2: Supplementary Table S2.** Associations of SLE unmet need definitions with mortality, adjusted for other potential confounding factors.

**Additional file 3: Supplementary Table S3.** Associations of SLE unmet need definitions with daily prednisolone dose (mg), adjusted for other potential confounding factors.

**Additional file 4: Supplementary Table S4.** Associations of SLE unmet need definitions with SF36-PCS, adjusted for other potential confounding factors.

**Additional file 5: Supplementary Table S5.** Associations of SLE unmet need definitions with SF36-MCS, adjusted for other potential confounding factors.

## Data Availability

The data underlining this article cannot be publically shared due to the strict protocols and procedures outlined in the Asia Pacific Lupus Collaboration (APLC) Data Access Policy to protect patients’ privacy and to maintain data security and ethical principles.

## Abbreviations

ACR: American College of Rheumatology
AMS: Adjusted mean SLEDAI
APLC: Asia Pacific Lupus Collaboration
BAFF: B cell activating factor
CI: Confidence intervals
GEE: Generalised estimating equations
HRQoL: Health-related quality of life
HDAS: High disease activity status
HR: Hazard ratio
IQR: Interquartile range
LLDAS: Lupus Low Disease Activity State
MCS: Mental component summary
MUHREC: Monash University Human Research Ethics Committee
PCS: Physical component summary
PGA: Physician Global Assessment
PWP-GT: Prentice, Williams and Peterson modelling with gap time
RA: Rheumatoid arthritis
SDI: SLICC-ACR Damage Index
SLE: Systemic lupus erythematosus
SLEDAI: SLE Disease Activity Index
SLICC: Systemic Lupus International Collaborating Clinics
TAM: Time-adjusted mean
