# Supplementary material for: ‘Not at target’: prevalence and consequences of inadequate disease control in systemic lupus erythematosus—a multinational observational cohort study
Source: Arthritis Res Ther. 2022 Mar 14;24:70. doi: 10.1186/s13075-022-02756-3 (PMC8919535; doi:10.1186/s13075-022-02756-3)
Supplement: Supplementary file 1 — Additional file 1: Supplementary Table S1. Associations of SLE unmet need definitions with organ damage accrual, adjusted for other potential confounding factors. [file 13075_2022_2756_MOESM1_ESM.docx]

**Supplementary Table S1** – Associations of SLE unmet need definitions with organ damage accrual, adjusted for other potential confounding factors

|  | **LLDAS-never** | **AMS>4** | **HDAS-ever** |
| --- | --- | --- | --- |
|  | **HR (95% CI), p-value** | **HR (95% CI), p-value** | **HR (95% CI), p-value** |
| **Damage accrual** | **1.46 (1.26,1.69), p<0.001** | **1.36 (1.16,1.59), p<0.001** | **1.81 (1.43,2.30), p<0.001** |
| Age at routine visit (years) | 1.02 (1.01,1.03), p<0.001 | 1.02 (1.01,1.03), p<0.001 | 1.02 (1.01,1.03), p<0.001 |
| Disease duration (years) | 1.01 (1.00,1.02), p=0.084 | 1.01 (1.00,1.02), p=0.071 | 1.01 (1.00,1.02), p=0.044 |
| Asian ethnicity | 0.60 (0.50,0.73), p<0.001 | 0.61 (0.50,0.74), p<0.001 | 0.61 (0.50,0.74), p<0.001 |
| Tertiary education | 0.86 (0.73,1.01), p=0.058 | 0.86 (0.74,1.01), p=0.069 | 0.87 (0.74,1.02), p=0.082 |
| Cumulative PNL exposure (g) | 1.04 (1.02,1.05), p<0.001 | 1.03 (1.02,1.05), p<0.001 | 1.03 (1.02,1.05), p<0.001 |
