# Supplementary material for: ‘Not at target’: prevalence and consequences of inadequate disease control in systemic lupus erythematosus—a multinational observational cohort study
Source: Arthritis Res Ther. 2022 Mar 14;24:70. doi: 10.1186/s13075-022-02756-3 (PMC8919535; doi:10.1186/s13075-022-02756-3)
Supplement: Supplementary file 2 — Additional file 2: Supplementary Table S2. Associations of SLE unmet need definitions with mortality, adjusted for other potential confounding factors. [file 13075_2022_2756_MOESM2_ESM.docx]

**Supplementary Table S2** – Associations of SLE unmet need definitions with mortality, adjusted for other potential confounding factors

|  | **LLDAS-never** | **AMS>4** | **HDAS-ever** |
| --- | --- | --- | --- |
|  | **HR (95% CI), p-value** | **HR (95% CI), p-value** | **HR (95% CI), p-value** |
| **Mortality** | **4.98 (2.07,12.0), p<0.001** | **2.36 (1.29,4.33), p=0.006** | **5.45 (2.75,10.8), p<0.001** |
| Cumulative PNL exposure (g) | 1.04 (1.00,1.08), p=0.033 | 1.05 (1.01,1.08), p=0.007 | 1.03 (1.00,1.07), p=0.047 |
| ACR/SLICC SDI score | 1.47 (1.29,1.68), p<0.001 | 1.50 (1.31,1.72), p<0.001 | 1.51 (1.32,1.72), p<0.001 |
