# Supplementary material for: ‘Not at target’: prevalence and consequences of inadequate disease control in systemic lupus erythematosus—a multinational observational cohort study
Source: Arthritis Res Ther. 2022 Mar 14;24:70. doi: 10.1186/s13075-022-02756-3 (PMC8919535; doi:10.1186/s13075-022-02756-3)
Supplement: Supplementary file 3 — Additional file 3: Supplementary Table S3. Associations of SLE unmet need definitions with daily prednisolone dose (mg), adjusted for other potential confounding factors. [file 13075_2022_2756_MOESM3_ESM.docx]

**Supplementary Table S3** – Associations of SLE unmet need definitions with daily prednisolone dose (mg), adjusted for other potential confounding factors

|  | **LLDAS-never** | **AMS>4** | **HDAS-ever** |
| --- | --- | --- | --- |
|  | **HR (95% CI), p-value** | **HR (95% CI), p-value** | **HR (95% CI), p-value** |
| **Prednisolone (mg/day)** | **5.71 (5.38,6.03), p<0.001** | **3.39 (2.95,3.83), p<0.001** | **9.04 (7.80,10.28), p<0.001** |
| Age at routine visit (years) | -0.07 (-0.08,-0.05), p<0.001 | -0.06 (-0.08,-0.04), p<0.001 | -0.08 (-0.10,-0.06), p<0.001 |
| Disease duration (years) | -0.09 (-0.11,-0.06), p<0.001 | -0.0 (-0.02,0.02), p=0.9 | -0.06 (-0.09,-0.04), p<0.001 |
| Asian ethnicity | 2.25 (1.72,2.78), p<0.001 | 1.77 (1.23,2.31), p<0.001 | 2.23 (1.67,2.79), p<0.001 |
| Presence of flare | 3.60 (2.83,4.38), p<0.001 | 5.19 (4.44,5.93), p<0.001 | 3.95 (3.29,4.61), p<0.001 |
| ACR/SLICC SDI score | 0.36 (0.17,0.55), p<0.001 | 0.29 (0.11,0.46), p=0.001 | 0.38 (0.19,0.56), p<0.001 |
