# Supplementary material for: ‘Not at target’: prevalence and consequences of inadequate disease control in systemic lupus erythematosus—a multinational observational cohort study
Source: Arthritis Res Ther. 2022 Mar 14;24:70. doi: 10.1186/s13075-022-02756-3 (PMC8919535; doi:10.1186/s13075-022-02756-3)
Supplement: Supplementary file 4 — Additional file 4: Supplementary Table S4. Associations of SLE unmet need definitions with SF36-PCS, adjusted for other potential confounding factors. [file 13075_2022_2756_MOESM4_ESM.docx]

**Supplementary Table S4** – Associations of SLE unmet need definitions with SF36-PCS, adjusted for other potential confounding factors

|  | **LLDAS-never** | **AMS>4** | **HDAS-ever** |
| --- | --- | --- | --- |
|  | **HR (95% CI), p-value** | **HR (95% CI), p-value** | **HR (95% CI), p-value** |
| **SF36-PCS** | **-1.40 (-1.71,-1.09), p<0.001** | **-0.96 (-1.50,-0.43), p<0.001** | **-2.17 (-2.78,-1.57), p<0.001** |
| Age at routine visit (years) | -0.10 (-0.13,-0.08), p<0.001 | -0.09 (-0.12,-0.06), p<0.001 | -0.10 (-0.13,-0.08), p<0.001 |
| Disease duration (years) | 0.06 (0.02,0.10), p=0.003 | 0.03 (-0.01,0.08), p=0.158 | 0.06 (0.02,0.10), p=0.003 |
| Asian ethnicity | 3.77 (2.72,4.82), p<0.001 | 4.62 (3.35,5.88), p<0.001 | 3.64 (2.60,4.69), p<0.001 |
| Tertiary education | 1.30 (0.74,1.86), p<0.001 | 1.43 (0.76,2.10), p<0.001 | 1.30 (0.74,1.86), p<0.001 |
| Cumulative PNL exposure (g) | -0.04 (-0.07,0.00), p=0.073 | -0.05 (-0.10,-0.01), p=0.027 | -0.05 (-0.09,-0.01), p=0.016 |
| Presence of flare | -1.11 (-1.50,-0.73), p<0.001 | -1.46 (-1.88,-1.03), p<0.001 | -1.15 (-1.54,-0.75), p<0.001 |
| Presence of organ damage | -3.01 (-3.53,-2.49), p<0.001 | -2.90 (-3.50,-2.31), p<0.001 | -2.96 (-3.48,-2.43), p<0.001 |
