# Supplementary material for: ‘Not at target’: prevalence and consequences of inadequate disease control in systemic lupus erythematosus—a multinational observational cohort study
Source: Arthritis Res Ther. 2022 Mar 14;24:70. doi: 10.1186/s13075-022-02756-3 (PMC8919535; doi:10.1186/s13075-022-02756-3)
Supplement: Supplementary file 5 — Additional file 5: Supplementary Table S5. Associations of SLE unmet need definitions with SF36-MCS, adjusted for other potential confounding factors. [file 13075_2022_2756_MOESM5_ESM.docx]

**Supplementary Table S5** – Associations of SLE unmet need definitions with SF36-MCS, adjusted for other potential confounding factors

|  | **LLDAS-never** | **AMS>4** | **HDAS-ever** |
| --- | --- | --- | --- |
|  | **HR (95% CI), p-value** | **HR (95% CI), p-value** | **HR (95% CI), p-value** |
| **SF36-MCS** | **-1.20 (-1.57,-0.84), p<0.001** | **-0.97 (-1.56,-0.38), p=0.001** | **-1.29 (-1.96,-0.63), p<0.001** |
| Asian ethnicity | 1.60 (0.46,2.73), p=0.006 | 1.47 (0.15,2.78), p=0.029 | 1.63 (0.50,2.77), p=0.005 |
| Tertiary education | 1.00 (0.34,1.66), p=0.003 | 1.13 (0.35,1.91), p=0.004 | 0.99 (0.33,1.66), p=0.003 |
| Cumulative PNL exposure (g) | 0.08 (0.04,0.12), p<0.001 | 0.04 (-0.02,0.09), p=0.178 | 0.08 (0.04,0.12), p<0.001 |
